# Unraveling the genetics of tomato fruit weight during crop domestication and diversification

**DOI:** 10.1007/s00122-021-03902-2

**Published:** 2021-07-12

**Authors:** Lara Pereira, Lei Zhang, Manoj Sapkota, Alexis Ramos, Hamid Razifard, Ana L. Caicedo, Esther van der Knaap

**Affiliations:** 1 Center for Applied Genetic Technologies, University of Georgia, Athens, GA USA; 2 Institute for Plant Breeding, Genetics and Genomics, University of Georgia, Athens, GA USA; 3 Biology Department, University of Massachusetts Amherst, Amherst, MA USA; 4 Department of Horticulture, University of Georgia, Athens, GA USA

## Abstract

**Key Message:**

Six novel fruit weight QTLs were identified in tomato using multiple bi-parental populations developed from ancestral accessions. Beneficial alleles at these loci arose in semi-domesticated subpopulations and were likely left behind. This study paves the way to introgress these alleles into breeding programs.

**Abstract:**

The size and weight of edible organs have been strongly selected during crop domestication. Concurrently, human have also focused on nutritional and cultural characteristics of fruits and vegetables, at times countering selective pressures on beneficial size and weight alleles. Therefore, it is likely that novel improvement alleles for organ weight still segregate in ancestral germplasm. To date, five domestication and diversification genes affecting tomato fruit weight have been identified, yet the genetic basis for increases in weight has not been fully accounted for. We found that fruit weight increased gradually during domestication and diversification, and semi-domesticated subpopulations featured high phenotypic and nucleotide diversity. Columella and septum fruit tissues were proportionally increased, suggesting targeted selection. We developed twenty-one F_2_ populations with parents fixed for the known fruit weight genes, corresponding to putative key transitions from wild to fully domesticated tomatoes. These parents also showed differences in fruit weight attributes as well as the developmental timing of size increase. A subset of populations was targeted for QTL-seq, leading to the identification of six uncloned fruit weight QTLs. Three QTLs, located on chromosomes 1, 2 and 3, were subsequently validated by progeny testing. By exploring the segregation of the known fruit weight genes and the identified QTLs, we estimated that most beneficial alleles in the newly identified loci arose in semi-domesticated subpopulations from South America and were not likely transmitted to fully domesticated landraces. Therefore, these alleles could be incorporated into breeding programs using the germplasm and genetic resources identified in this study.

**Supplementary Information:**

The online version contains supplementary material available at 10.1007/s00122-021-03902-2.

## Introduction

Domestication of crop plants has led to an increase in phenotypic variation, especially for certain traits (Meyer and Purugganan 2013). Collectively called the “domestication syndrome,” traits that ease the harvest, lead to improved nutritional value and lead to growth under managed conditions were targets of selection in most modern day crops. These domestication traits are considered to have benefitted the growth of agrarian societies and urbanization. Yet many other traits, such as resistance to biotic and abiotic stress as well as certain quality traits, may have been lost as selection ensued. In cereals such as maize, rice and barley, the most selected traits were plant and inflorescence structure, shattering and other seed traits (Gross and Olsen 2010). Knowledge about the domestication and diversification history of these cereals has helped breeders to identify germplasm that may harbor traits that can be employed in crop improvement programs.

Contrary to staple crops, the domestication process is not as well understood in fruit and vegetable crops such as tomato. While recent studies have provided insights into the genetic history of tomato (Blanca et al. 2015; Razifard et al. 2020), the selection of traits in this vegetable appeared to be less linear. Vegetables and fruit tend to be consumed for their nutritional quality. such as vitamin and mineral content and less for the caloric intake (Slavin and Lloyd 2012). It is generally assumed that fruit weight was an important selection criterion for most vegetables and fruits, but whether this trait was selected as extensively as grain size and yield in cereals is less clear. It is therefore possible that certain alleles affecting vegetable weight and yield may not have been incorporated into these crops as a result of the prioritization for other traits with cultural and/or culinary importance to local populations. In contrast, most domestication traits in staple crops are nearly fully fixed in modern germplasm (Gross and Olsen 2010).

Different hypotheses have been proposed to explain tomato’s domestication and diversification trajectory. One of them provides a two-step process from the fully wild and red-fruited *Solanum pimpinellifolium* (SP) to the semi-domesticated *Solanum lycopersicum* var. *cerasiforme* (SLC), and a second step from SLC to the fully domesticated *Solanum lycopersicum* var. *lycopersicum* (SLL) (Lin et al. 2014; Blanca et al. 2015). An alternative hypothesis states that SLC diverged from SP as a fully wild species. Later, human selection gave rise to domesticated SLC populations that have largely replaced the wild SLC (Razifard et al. 2020). Further insights from these studies could lead to the identification of subpopulations that feature large genetic diversity for the identification of novel and beneficial alleles for crop breeding.

Thus far, just three loci controlling tomato fruit weight are cloned: *fw2.2* (Frary et al. 2000), *fw3.2* (Chakrabarti et al. 2013) and *fw11.3* (Mu et al. 2017). An additional two loci that control locule number affect weight as well: *lc* (Muños et al. 2011) and *fas* (Cong et al. 2008; Xu et al. 2015). Together these five genes represent the three main processes that define tomato fruit weight: 1. floral meristem enlargement and organization; 2. cell proliferation (division) and 3. cell expansion (van der Knaap et al. 2014). The cell proliferation stage may extend longer or shorter and faster or slower as well as in anticlinal and/or periclinal directions. Similarly, the cell expansion stage may extend longer and cells can expand in different directions. In addition, in complex organs such as the fruit, one tissue type may expand more than another, and the developmental timing when these processes are altered may also differ. Modifications of these processes impact the weight of the fruit as well as its overall morphology.

The most studied tissue type in terms of growth and cellular pattern in tomato fruits is the pericarp (Cheniclet et al. 2005; Renaudin et al. 2017). In the cherry tomato line Wva106, cell layers are nearly determined in flowers when female meiosis starts, while cell volume continuous to increase until anthesis (Renaudin et al. 2017). As soon as one day after anthesis, the mitotic activity in the pericarp resumes, consisting of periclinal and anticlinal cell divisions to generate, respectively, more cell layers and more cells in a given layer. Cell volume increases during the first stage of fruit growth but then increases exponentially 7–14 days after anthesis eventually reaching a final size that is approximately 170-fold larger than ovary pericarp cells in Wva106. This study showed that the number of cell layers and the cell area at anthesis were comparable among 20 different tomato varieties. On the other hand, twofold and sixfold differences were observed at the breaker stage for cell layers and cell size, respectively (Cheniclet et al. 2005). This implies that cell size may be an important driver of tomato fruit weight.

With respect to the organization of the floral meristem in tomato, *SlWUSCHEL* (Muños et al. 2011) and *CLV3* (Xu et al. 2015) are the main regulators. They act in a negative feedback loop to affect meristem size in a pathway that is highly conserved in plants (Schoof et al. 2000; Somssich et al. 2016; Liu et al. 2021). The deregulation of the tomato WUS-CLV pathway causes changes in floral organ number, resulting in fruits with three or four locules instead of two, and a subsequent increase in fruit weight (Xu et al. 2015; Chu et al. 2019). A similar impact is noted in corn where the manipulation of *CLE7* expression by CRISPR-Cas9 editing of the promoter leads to higher yielding lines (Liu et al. 2021). With respect to cell proliferation, two of the cloned fruit weight genes, *Cell Number Regulator* (*CNR/FW2.2*) and *SlKLUH* (*FW3.2*), have been shown to alter cell divisions (Frary et al. 2000; Chakrabarti et al. 2013). With respect to cell enlargement, only one gene, *Cell Size Regulator* (*CSR/FW11.3*), is known to control this trait (Mu et al. 2017).

Many fruit weight QTL have been identified in various studies (Eshed and Zamir 1995; Grandillo et al. 1999; Causse et al. 2002; Illa-Berenguer et al. 2015; Barrantes et al. 2016), indicating a complex genetic architecture of the trait. Therefore, it is likely that many other hitherto unknown genes were selected during tomato’s evolution. The discovery of these additional selection loci could allow for a better understanding of the genetic basis controlling organ weight and the genomic regions that were selected during tomato’s recent evolution.

In this study, we analyzed native accessions of SP, SLC and SLL from South and Central America to explore the phenotypic variation for fruit weight and weight-related traits with the goal to identify novel loci controlling this trait. Considering the narrow genetic diversity observed in the cultivated germplasm, the aim is to discover improvement alleles that may have been left behind during the early selection of tomato and could be incorporated into modern varieties. The inclusion of a broader sampling of SLC and SLL landraces and the detailed characterization of developmental processes contributing to fruit weight should lead novel insights into this critical area of fruit and vegetable research.

## Materials and methods

### Plant material

The Varitome collection comprised of 28 wild SP, 117 semi-domesticated SLC and 21 ancestral SLL accessions (previously described in Razifard et al. 2020; SGN https://solgenomics.net/) was used in this study. The collection was grown in three replicates in 2016 at Live Oak, FL and two field sites in Athens, GA, with three plants of each accession per replicate. Average fruit weight of each accession was highly correlated in all replicates (Supplementary Fig. 1A). A subset of 33 SP and SLC accessions that represented the genetic diversity in the Varitome collection was grown at Live Oak, FL again in 2017. Both fruit weight and morphological phenotypes were highly correlated between the 2016 and 2017 replicates (Supplementary Fig. 1B). All plants were grown following standard tomato farming practices and were watered using drip irrigation.

F_1_ plants were generated by manually crossing the selected accessions grown in the greenhouse in Athens, GA. F_1_ seedlings were genotyped to confirm the cross with at least one molecular marker (Supplementary Table 1) and self-pollinated to obtain F_2_ seeds. F_2_ seedlings were germinated in a controlled environment and transplanted to the field to evaluate fruit weight. Twenty-one F_2_ populations and their parental accessions were grown in four field sites (Athens, GA; Vidalia, GA; Blairsville, GA; and Live Oak, FL) from 2017 to 2019 (www.solgenomics.net for detailed information). F_3_ seeds were harvested from the mature fruits of field grown F_2_ plants to perform progeny tests. F_3_ seedlings were genotyped at targeted genomic regions, and 10 homozygous plants of each genotype were grown in the field and evaluated.

### Phylogeny construction and diversity parameters

A phylogenetic tree was built using the coalescent-based SVDquartets method (Chifman and Kubatko 2014) implemented in PAUP (v. 4a157) (Swofford 2003) based on 69,163 fourfold-degenerate (4D) SNPs obtained in Razifard et al. (2020), excluding SNPs missing in > 10% of all accessions. The number of quartets was fixed to *n*^*3*^, where *n* is the number of accessions (166). Three accessions were re-classified according to the previous phylogenetic and population structure results (Razifard et al. 2020) as well as their phenotypic and geographical descriptions (SGN https://solgenomics.net/). The SP from Northern Ecuador BGV006148 and BGV006230 exhibited plant architecture different from a typical SP plant, and their fruit weight was much larger than the average SP fruit weight; therefore, the two accessions were re-classified as admixture SLC and Ecuadorian SLC, respectively. The third accession SLL BGV008106 was re-classified as Mexican SLC because its fruit weight was much smaller than a typical SLL plant. These reassignments are also consistent with a previous study (Razifard et al, 2020).

Average nucleotide diversity (*π*) was calculated using diploid sample sizes and for non-overlapping 10-kb windows using VCFtools (v0.1.15) (Danecek et al. 2011). Watterson's theta (*Θ*_*W*_) was calculated in R based on sample size, number of SNPs and SNP density.

### Phenotyping of fruit weight and related traits

Fruit weight was measured by collecting approximately 40 fruits from each plant. Twenty representative fruits were bulk-weighed using a VWR-3001E top loading balance or a VWR-64B analytical scale. Average fruit weight was recorded for each plant. The fruit weight of the Varitome accessions for this research was the average value of three field replicates.

In addition to fruit weight, 11 fruit weight-related traits were phenotyped, including locule number, tissue areas and pericarp cell layer, cell size and circumference cell number (Supplementary Table 2, Supplementary Fig. 2). Locule number was counted in 40 fruits per accession. Morphological traits were analyzed using the scanned images of fruit cross sections. About eight fruits per accession were sliced along the medio-lateral axis at the equatorial plane, and one of the halves was scanned at 300 dpi using an HP Scanjet G4050. All fruit images were analyzed using the software Tomato Analyzer 4.0 (Rodríguez et al. 2010) to obtain tomato fruit area, pericarp area, septum area and columella area. The area ratio of pericarp, septum and columella was calculated by dividing the area of respective tissue by the total area. Nine accessions were excluded from the morphological analysis due to their high locule number and/or irregular shapes. The cellular components of pericarp tissue were measured in 0.5–1-mm-thick slices. For each accession, four fruits at breaker or mature green stage were used. Three pericarp slices were collected by hand from the equatorial region along the proximal–distal axis in each fruit using double-edge razor blades. The pericarp slices were stained in 0.15% toluidine blue solution for five seconds and then rinsed with water. Images of the stained slices were taken by Olympus DP70 camera mounted on an OLYMPUS MVX10 optical microscope using an Olympus MVX-TVO.63XC adapter. The pericarp thickness, cell layer and maximum cell size were measured from the microscopy images using the software ImageJ bundled with 64-bit Java 1.8.0_112 (Schneider et al. 2012) and the MorphoLibJ plugin (Legland et al. 2016). The number of cells along the fruit circumference was estimated by two methods as described in Supplementary Table 2. The two circumference cell number calculated in both methods correlated with each other (Supplementary Fig. 1C), so only circumference cell number 2 was used in follow-up studies.

The ovary pericarp cellular traits were phenotyped in 33 accessions grown in Live Oak, FL in 2017. Approximately 10 flowers at anthesis stage were collected from each plant; the petals and sepals were removed and the exposed ovaries were halved along the proximal distal axis. One of the halves was fixed in the FAA solution (50% ethanol, 10% 37% formaldehyde, 5% glacial acetic acid) in a vaccum chamber for one hour. The fixed ovaries were dehydrated in an ethanol solution with gradually increasing concentration (50%, 70%, 85%, 95% and 100% twice) with 30–60 min gentle shaking in each solution and then rehydrated in ethanol solution with decreasing concentration (95%, 85%, 70%, 50%, 30%, 15% and water twice). The ovaries were then stained in 10 μg/ml propidium iodide solution for 1 h and washed with water before dehydrated with ethanol solution as in the previous step. In the last step, ovaries were cleared in a 1:1 solution of ethanol:methyl salicylate for 2 h and kept in methyl salicylate at 4 °C for imaging. At least five ovaries were imaged for each accession using Zeiss LSM 880 Confocal Microscope in the Biomedical Microscopy Core at the University of Georgia, and the ovary pericarp cellular traits were measured in ImageJ (Supplementary Table 2, Supplementary Fig. 2).

### Genotyping for known fruit weight genes and novel fruit weight QTLs

Genomic DNA used for genotyping was extracted from young leaves at seedling stage using CTAB extraction buffer followed by chloroform purification (Doyle 1991). Known fruit weight genes and the novel fruit weight QTLs were genotyped using markers described in Supplementary Table 1.

### QTL-Seq experiment and analysis

The DNA of F_2_ plants was extracted from young leaves at the seedling stage using the Qiagen’s DNAeasy 96 Plant Kit following the manufacturer’s instruction. In each population, DNA from ten plants with highest (large bulk) and lowest (small bulk) fruit weight was quantified using a Qubit 2.0 Fluorometer to add an equal quantity of DNA from each plant in the two bulks. Library preparation was done using the NEBNext Ultra DNA Library Prep kit, and the sequencing was done in one lane of the Illumina NextSeq high output flowcell (PE150, 300 479 cycles) at the Georgia Genomics and Bioinformatics Core at University of Georgia, Athens. The resulting sequences were processed and mapped to the tomato reference genome (version SL3.0) using Burrows Wheeler Aligner-MEM (Li et al. 2009; Li 2013), Picard Tools (http://broadinstitute.github.io/picard/) and Genome Analysis Toolkit (GATK) (McKenna et al. 2010) with the recommended default settings. SNP variants were called using GATK Haplotype caller and filtered with the recommended default settings. The QTL-seq analysis was conducted using the QTLseqr R package (Mansfeld and Grumet 2018). The VCF files were filtered, and only SNP with quality of higher than 40 was kept. The read depth at each SNP in each bulk was at least 4, but no more than 90 when considering both bulks together. Window size was set to 1 Mb and 10,000 simulations were run creating simulated deltaSNP ﻿indexes based on the data parameters. The simulations extreme quantiles served as confidence intervals for the absolute value of ΔSNP, which were plotted as the QTL-seq final results using ggplot2 package (Wickham 2011). QTLs above the 95% confidence interval were considered significant and investigated further.

### Statistical analysis and QTL mapping

All statistical analyses were performed in R unless specified otherwise. The principal component analysis (PCA) was performed using the subset of 147 accessions without missing data with the function prcomp. Shapiro–Wilk test was used (function shapiro.test) to determine whether the fruit weight for each F_2_ population fit normal distribution; if not, the fruit weight was normalized using the quantile normalization function (function qqnorm). In the three F_2_ populations selected for QTL-seq analysis (17S62, 18S133 and 18S40), QTL regions identified were genotyped in each plant in all three populations with at least three KASP markers (Supplementary Table 1). The genetic maps were constructed using R package R/qtl (Broman et al. 2003) with the Kosambi function to estimate genetic distances. Composite interval mapping analysis was performed using the same package with a permutation test (*n* = 1000) to determine the significance threshold. QTLs above the 95% confidence interval were considered significant. Additional F_2_ populations were genotyped with markers in the QTLs regions that were confirmed by progeny testing in the QTL-seq populations. ANOVA was used to assess the association of the F_2_ fruit weight and genotypes at the loci.

## Results

### Fruit weight and weight-related traits of Varitome collection

**Fig. 1:**
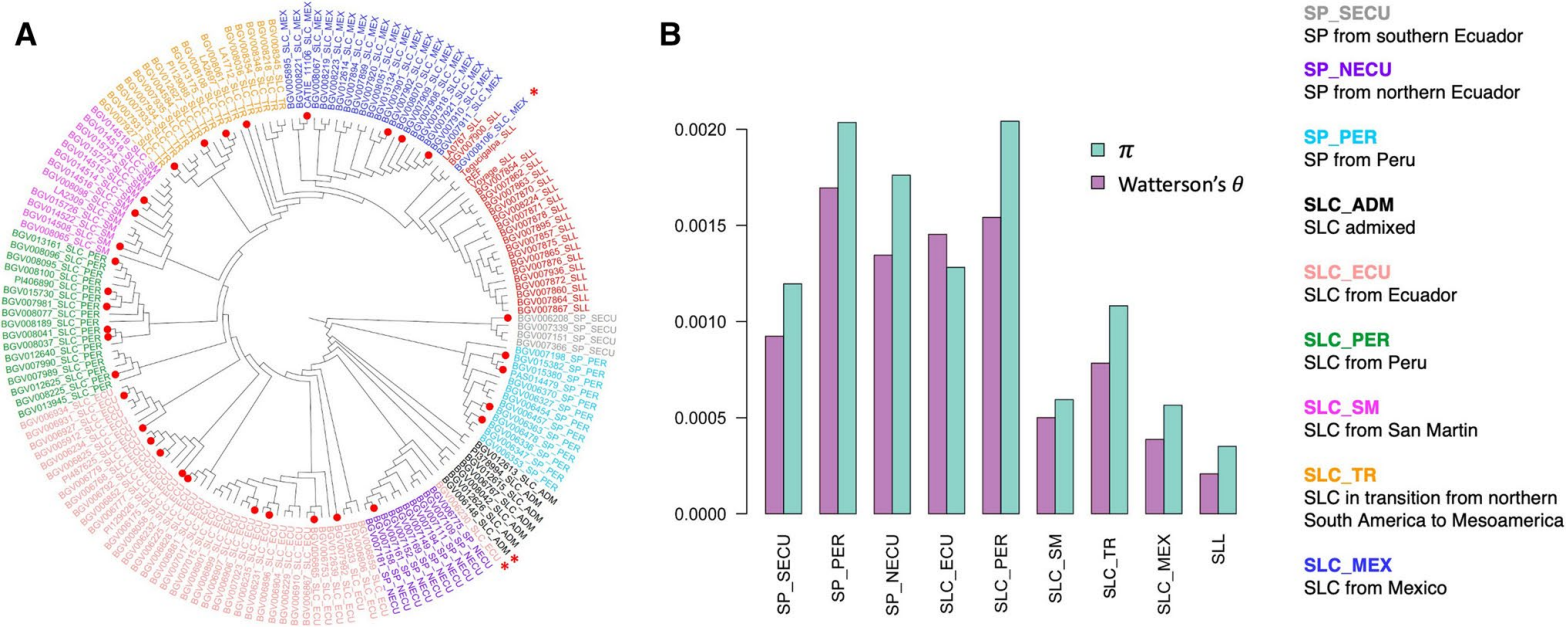
**A.** Phylogenetic tree using genome-wide fourfold-degenerate SNPs after quality filtering. Population group is represented by color and indicated at each accession. Population delimitation of three accessions (*) was manually corrected according to their physical traits and previous phylogenetic study (Razifard et al, 2020). Accessions representing diversity of SP and SLC populations (red dot at tree tip) were selected for in-depth fruit phenotyping. **B**. Nucleotide diversity in each population, estimated based on π and Watterson's θ in 100-kb genomic windows

The Varitome collection represents accessions that span the evolution from fully wild to the earliest tomato domesticates (Razifard et al, 2020). The phylogenetic relationship of the accessions showed that SP, the wild ancestor of cultivated tomato, were separated into three subpopulations (Fig. 1A). These subpopulations corresponded to different geographical origins: Southern Ecuador (SP_SECU), Peru (SP_PER) and Northern Ecuador (SP_NECU) and showed high genetic diversity (Fig. 1B). The SLC accessions arose most likely from SP_NECU and were separated into South American and transitional subpopulations, the latter resulting from the northward spread of the South American SLC (Razifard et al, 2020) and eventually gave rise to the cultivated SLL. The South American SLC_ECU and SLC_PER showed high levels of genetic diversity, comparable to SP (Fig. 1B). The transitional subpopulation SLC_TR included accessions from a wide geographical distribution, from Colombia to northern Mexico, and showed a higher genetic diversity than the more geographically confined subpopulations SLC_SM and SLC_MEX (Fig. 1B). In Mexico, SLC_MEX likely gave rise to the ancestral SLL, a process that was accompanied by a substantial loss in genetic diversity as is a common consequence of domestication. Because of the diversity span in the Varitome collection, the fruit weight variation should accurately reflect the change of this trait through the evolution of tomato.

**Fig. 2:**
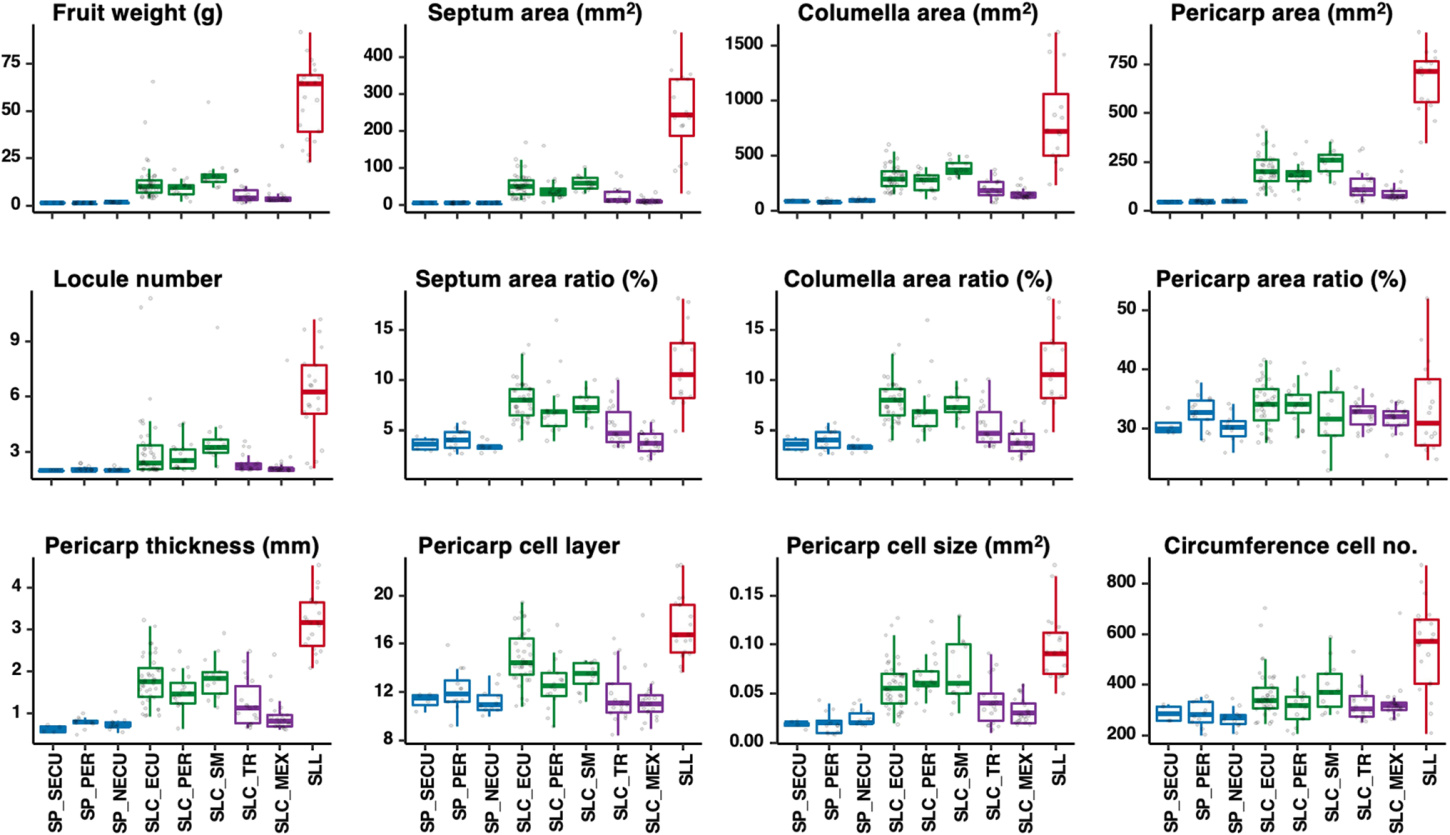
Fruit weight and weight-related traits of the Varitome accessions grouped by the subpopulations. The wild SP subpopulations, South American SLC, transitional SLC subpopulations and the SLL population are colored in blue, green, purple and red, respectively

The Varitome collection (*n* = 166) was phenotyped for fruit weight and weight-related traits, except for the admixed accessions (SLC_ADM). We observed a dramatic increase in fruit weight from SP to SLL (Fig. 2). Not surprisingly, the SP accessions in all subpopulations bore small fruits weighing on average 1.5 g, ranging from 0.9 to 2.0 g. Large increases in fruit weight were observed in the South American SLC subpopulations closest related to SP. These accessions bore fruits ranging from 2 to 65 g, weighing on average 13 g. The transitional SLC accessions bore smaller fruits with an average fruit weight of 5.6 g. SLL accessions bore the largest fruits, weighing 56 g on average and ranging from 23 to 91 g. The change in locule number presented a similar trend as fruit weight (Fig. 2), with an average of two in SP, three in SLC subpopulations and six in SLL.

To evaluate the area of the major fruit tissues, e.g., pericarp, septum and columella, scanned cross sections of all fruits were analyzed using Tomato Analyzer (Supplementary Fig. 2 and Fig. 2). The area measurements for each tissue type showed high correlation with fruit weight (Supplementary Fig. 3), indicating that these increases mirrored the weight increase evolving in tomato. To determine whether certain tissue types increased proportionally more than others, we divided the respective area over the total cross-sectional area to obtain the ratio. The pericarp enlarged proportionally to the increase of the entire fruit area such that the pericarp area ratio remained similar in all subpopulations. In contrast, the septum area ratio doubled to 8% in the South American SLC compared to SP and increased further to 11% in SLL. The columella area ratio increased slightly from 6% in SP to 9% in SLC and increased more dramatically to 17% in SLL. The disproportional increase in the septum and columella areas suggested that these tissues were targeted for enlargement during tomato domestication. We further analyzed the pericarp in detail by measuring the pericarp thickness and two major components of thickness: cell size and cell layer (Fig. 2). The average pericarp thickness increased from 0.8 mm with 11–12 cell layers in SP to 1.8 mm with 14 cell layers in the South American SLC accessions. SLL showed the highest pericarp thickness averaging at 3 mm and 17 layers. The increase in maximum pericarp cell size presented a similar trend as the other weight-related traits, ranging on average from 0.02 mm^2^ in SP to 0.09 mm^2^ in SLL population. Based on cell size from the microscopy images and the average fruit perimeter value of each accession as measured by Tomato Analyzer, we calculated the cell number in the circumference of the fruit. This calculation estimated the growth along the medio-lateral axis. The average circumference cell number was approximately 280 in the SP to 380 in South American SLC. A dramatic increase in circumference cell number was found in SLL, with an average of 540. For nearly all the weight and weight-related traits, the SLL showed the largest range, followed by SLC_ECU.

**Fig. 3:**
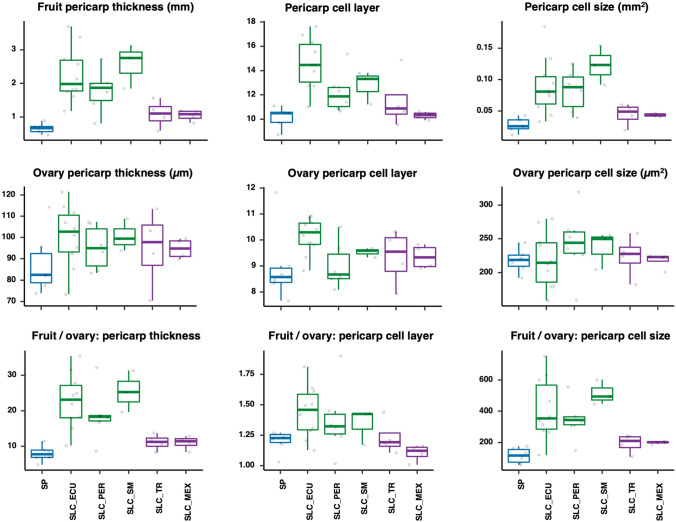
Mature fruit and ovary pericarp phenotypes in selected SP and SLC accessions (red dot at tree tip in Fig. 1). The wild SP subpopulation, South American SLC and transitional SLC subpopulations are colored in blue, green and purple, respectively

The data suggested that selection for increased fruit weight during domestication and diversification of the species targeted the columella and septum tissues. This could drive growth in the medial–lateral direction as exemplified by the large increase in the pericarp circumference cell number in SLL. To further evaluate whether selection acted on the development of the ovary in the flower or on the growth of the fruit after pollination or both, we compared the pericarp weight-related traits in the ovary and mature fruit on a subset of the population. We randomly selected SP and SLC accessions that represented the genetic diversity in the Varitome collection. At anthesis, the SLC subpopulations had thicker ovary pericarps than SP (Fig. 3). The higher ovary pericarp thickness was mostly due to the increase in cell layers, since the cell size varied less on average among the subpopulations. During fruit development, the cell layers increased modestly by, on average, 1.2 times in SP to 1.4 in SLC_ECU. A more dramatic increase was noted for fruit pericarp cell size after anthesis. Between the ovary and the fruit pericarp, cell size increased from on average 130 times in SP to 350–500 times in South American SLC. This finding suggested that the dramatic increase in fruit weight during the evolution from SP to South American SLC was a combination of increased cell division in the ovary pericarp during floral development and increased cell expansion in the fruit pericarp during fruit development. In contrast, the fruit weight difference between South American and transitional SLC subpopulations was primarily due to differences during fruit development. Most of the fruit weight-related traits in mature fruits correlated with weight and with each other (Supplementary Fig. 4A). On the other hand, the ovary pericarp traits were less correlated to the mature fruit traits (Supplementary Fig. 4B). The principal component analysis (PCA) using fruit weight and eleven weight-related traits showed that the Varitome accessions clustered according to their subpopulations (Supplementary Fig. 5). The SP fruits were homogeneous, whereas the SLL fruits were highly variable and distinct from SP. The South American SLC exhibited fruit trait values that were intermediate between SP and SLL on both PCs, yet the transitional SLC showed more overlap with the SP accessions. Most traits, except for pericarp area ratio, contributed equally to the variance explained by PC1. The pericarp area ratio, thickness, cell size and circumference cell number traits contributed primarily to PC2.

### Known fruit weight genes in the Varitome collection

**Fig. 4:**
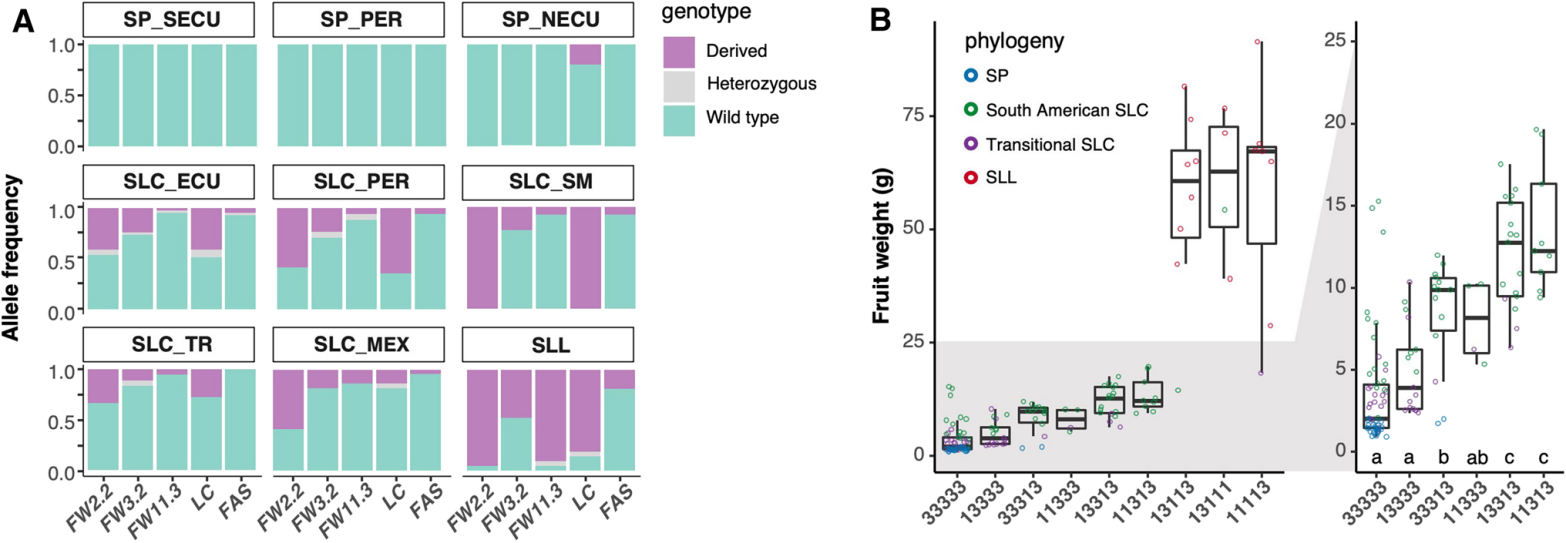
**A.** Frequency of the derived and wild-type alleles for the known fruit weight loci in each subpopulation. **B.** Fruit weight of accessions grouped by the genotypic combination of known fruit weight loci: *fw2.2*, *fw3.2*, *fw11.3*, *lc* and *fas*; the five-digit number denotes the genotype of the five loci in this order. Number 1 represents derived alleles found in the cultivated tomatoes, and 3 represents the wild-type alleles found in wild tomatoes. Only groups with more than three accessions are presented. The letters at the bottom of each box plot represent significance groups as determined by the Tukey HSD test

The genetic control of fruit weight is partially understood in tomato (van der Knaap et al. 2014). To investigate when the alleles arose, we genotyped the Varitome collection for *fw2.2*, *fw3.2*, *fw11.3*, *lc* and *fas* using markers distinguishing the derived from the wild-type (WT) alleles. As expected, nearly all SP accessions were fixed for the WT allele at the five fruit weight loci (Fig. 4A). The exceptions were found in the closest relatives of SLC: two SP_NECU that carried derived allele of *lc*. The derived alleles of the other fruit weight genes seem to have arisen in SLC_ECU. The derived allele of *fw11.3* and *fas* was rare in the SLC subpopulations. In SLL, the frequency of *fas* remained low at 20%, whereas *fw11.3* became almost fixed at 93%. The *lc* allele frequency was only 10% in SLC_MEX before increasing to 85% in the SLL population in the Varitome collection. The allele frequency of *fw2.2* increased from 30% in SLC_TR to 60% in SLC_MEX and became nearly fixed in SLL. The derived allele of *fw3.2* remained relatively low in all SLC, before increasing to 45% in the SLL population. These findings suggested that *lc*, *fw2.2* and *fw11.3* were the most important fruit weight genes in the final stages of tomato domestication.

To investigate the contribution of the known genes on fruit weight, we grouped the Varitome accessions based on the allelic combinations at the loci. We do not show all genotypic combinations, since the derived allele of several loci was underrepresented in the collection. In general, the accumulation of the derived alleles correlated with an increase in fruit weight (Fig. 4B). The accessions that carried two or more derived alleles of the fruit weight loci were significantly larger than those carrying the WT allele for all loci. Interestingly, the derived allele of *fw2.2* had a limited effect on fruit weight in this population. A great increase in fruit weight was found in accessions that carried the derived allele of *fw11.3*, most of which were SLL. Nevertheless, the known fruit weight genes partially accounted for the variation in weight. Among accessions carrying the WT alleles at all five loci, fruit weight varied from 1.2 to 15 g, with the SPs carrying the smallest fruits and the SLCs carrying the largest fruits. Similarly for allelic combinations with more derived alleles (11113), fruit weight varied from 16 to 87 g. This large phenotypic variation within a genetic group suggested additional genetic components that could be mined from this diverse and ancestral tomato collection.

### Mapping of novel fruit weight loci in bi-parental populations

To search for additional loci that contribute to tomato fruit weight, we created bi-parental populations that bridge important evolutionary transitions: the initial step from SP to SLC and the second step from SLC to SLL. We selectively crossed accessions with different fruit weights while carrying the same allele at the five known weight loci, in order to avoid mapping the same genes (Supplementary Table 3). Nine F_2_ populations were developed between SP and SLC_ECU, which was the SLC subpopulation most related to SP. Only one F_2_ population was developed between SLL and SLC due to the difficulty in finding SLC accessions with multiple derived alleles. Furthermore, six populations were developed between accessions from the larger-fruited South American SLC and smaller-fruited transitional SLC subpopulations. In general, the F_2_ populations showed an asymmetric fruit weight distribution, biased toward the small-fruited parent, but the skewness varied among the populations (Supplementary Figs. 6–7). The strongest bias toward the small-fruited parent occurred in the SP x SLC_ECU populations (Supplementary Fig. 6), where in most cases, the largest F_2_ fruits still weighted approximately half that of the large-fruited parent. The populations developed among the SLC or between SLC and SLL showed less skewed fruit weight distribution (Supplementary Fig. 7), with the exception of population 17S62. The strong bias toward the small-fruited parent in 17S62 resembled that of the SP x SLC_ECU populations. Contrary to the SLC x SLC and SLC x SLL populations, the larger genetic distance between the SP and SLC accessions and the skewed fruit weight distribution in the F_2_ toward the small-fruited parent, more QTLs of variable effects were expected to segregate in the wider crosses. In addition to the populations spanning important evolutionary transitions in tomato, we developed five F_2_ populations with accessions from closely related subpopulations. In most cases, the fruit weight was normally distributed between the parental accessions (Supplementary Fig. 8) with evidence of transgressive segregation. The lower genetic variation between the parents of these segregating populations and the normal, non-skewed distribution of fruit weight suggested fewer QTLs contributing to the trait.

### Identification of novel fruit weight QTLs by QTL-seq analysis

**Fig. 5:**
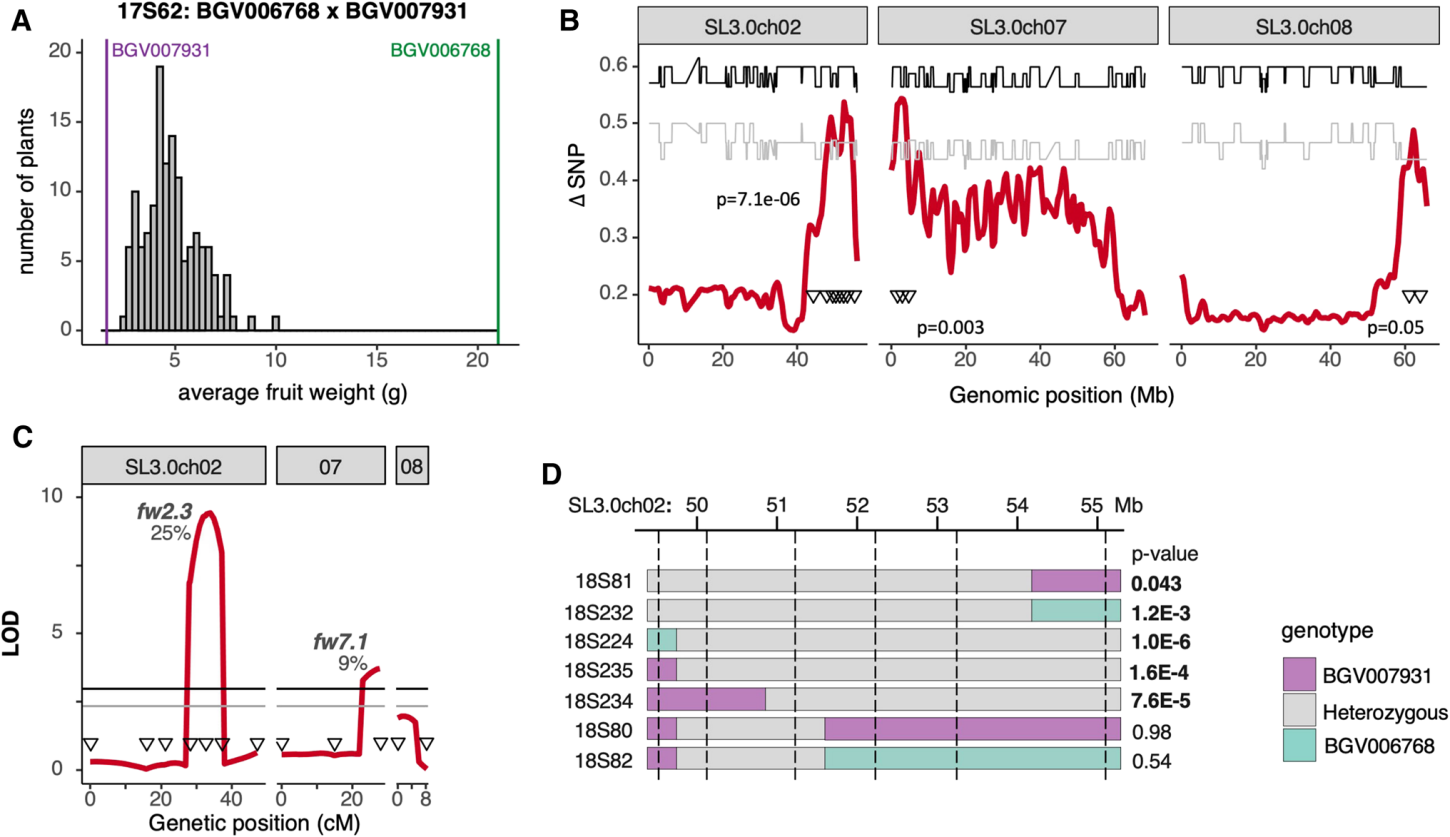
**A.** Fruit weight distribution of F_2_ plants in population 17S62. The vertical bars represent the fruit weight of parental accessions grown together with the F_2_ population. **B.** Fruit weight QTL-seq analysis of 17S62. Average ΔSNP values (red) in the 1 Mb sliding window are shown. Confidence interval at 95% (gray) or 99% (black) is represented in each plot. Genetic markers (triangles) were used to validate the QTL in the entire population. The p-values represent the correlation between the fruit weight and the most associated markers. **C.** Composite interval mapping of fruit weight in 17S62 using the indicated markers (triangles). LOD score (red) and confidence interval at 95% (gray) or 99% (black) are represented. The percentage of trait variation accounted by each significant QTL is shown. **D.** Fruit weight of F_3_ progenies segregating at the heterozygous regions was compared by Student’s *t* test. Dotted vertical lines mark the position of markers

We selected three F_2_ populations that most likely would identify distinct loci from the QTL-seq analysis: 17S62, 18S133 and 18S40. The assessment was based on whether the parental fruits showed that the increase might be predominantly due to cell layer or cell size, and whether the pericarp or columella areas disproportionally increased the most. Further, we determined the developmental timing of the fruit weight differences between parents, to be primarily during ovary or during fruit development. The first population selected, 17S62 (*n* = 132), was derived from parents that carried the WT allele for all known fruit weight loci and presented the largest fold difference in the fruit weight among accessions with the same known fruit weight alleles (Fig. 5A). The parents were the SLC_ECU BGV006768 and SLC_TR BGV007931 carrying fruits with an average weight of 21 g and 1.6 g, respectively. The pericarp area ratio was 50% in BGV006768 and 30% in BGV007931 (Table 1A). The larger pericarp in BGV006768 was due to a combination of more cell layers and larger cell size. In addition, the difference in weight appeared to be predominantly determined by more cell enlargement in BGV006768 during fruit development. The fruit weight QTL-seq analysis resulted in three new QTLs at the bottom of chromosome 2 (*fw2.3*), the top of chromosome 7 (*fw7.1*) and the bottom of chromosome 8 (*fw8.1*) (Fig. 5B, Supplementary Fig. 9A). Markers covering the QTL intervals were developed to genotype the entire F_2_ population. Both *fw2.3* and *fw7.1* were validated with composite interval mapping using entire F_2_ population; they each accounted for 25% and 9% of fruit weight variation in 17S62, respectively (Fig. 5C, Supplementary Table 4). We further conducted progeny testing in F_3_ families segregating around *fw2.3* to confirm the QTL. The increase in fruit weight came from the SLC_ECU BGV006768. The F_3_ seedlings were genotyped and fruit weight of homozygous plants carrying contrasting genotype was compared in each family. *fw2.3* was confirmed in eight F_3_ progeny families and was delimited to an interval between 51.3 Mb and 55.3 Mb, consistent with the composite interval mapping result (Fig. 5D, Supplementary Table 5).

**Table 1:** Fruit phenotypes of parental accessions in F_2_ populations

*A. 17S62: BGV006768 vs BGV007931*							
Mature fruit cross section				Pericarp cellular measurement			
	BGV006768	BGV007931			BGV006768	BGV007931	Ratio^*^
Total area (mm^2^)	1070.6	131.0	Ovary pericarp	Thickness (mm)	0.11	0.07	1.52
Pericarp (mm^2^)	534.7	42.8		Cell layer	10.9	7.9	1.37
Columella (mm^2^)	77.6	10.6		Max. cell size (µm^2^)	213	183	1.16
Septum (mm^2^)	44.1	5.4					
Pericarp/total area (%)	49.9	29.2	Mature fruit pericarp	Thickness (mm)	3.39	0.59	5.78
Columella/total area (%)	7.2	8.1		Cell layer	16.33	9.58	1.71
Septum/total area (%)	5.1	4.1		Max. cell size (µm^2^)	134,017	19,929	6.72
*B. 18S133: BGV006225 vs BGV007181*							
Mature fruit cross section				Pericarp cellular measurement			
	BGV006225	BGV007181			BGV006225	BGV007181	Ratio*
Total area (mm^2^)	473.3	170.8	Ovary pericarp	Thickness (mm)			
Pericarp (mm^2^)	167.6	44.3		Cell layer			
Columella (mm^2^)	33.3	12.8		Max. cell size (µm^2^)			
Septum (mm^2^)	32.4	4.5					
Pericarp/total area (%)	35.4	26.0	mature fruit pericarp	Thickness (mm)	1.53	0.55	2.78
Columella/total area (%)	7.0	7.5		Cell layer	14.44	10.85	1.33
Septum/total area (%)	6.8	2.6		Max. cell size (µm^2^)	46,017	15,713	2.93
*C. 18S40: BGV008041 vs PI406890*							
Mature fruit cross section				Pericarp cellular measurement			
	BGV008041	PI406890			BGV008041	PI406890	Ratio*
Total area (mm^2^)	412.2	413.1	Ovary pericarp	Thickness (mm)	0.08	0.08	1.01
Pericarp (mm^2^)	169.2	140.1		Cell layer	8.1	8.5	0.95
columella (mm^2^)	38.5	50.8		Max. cell size (µm^2^)	236	159	1.48
septum (mm^2^)	27.8	20.8					
pericarp/total area (%)	41.1	33.9	Mature fruit pericarp	Thickness (mm)	2.72	1.40	1.95
columella/total area (%)	9.3	12.3		Cell layer	15.36	12.33	1.25
septum/total area (%)	6.7	5.0		Max. cell size (µm^2^)	82,500	48,517	1.70

^*^Ratio between the large-fruited and small-fruited values

**Fig. 6:**
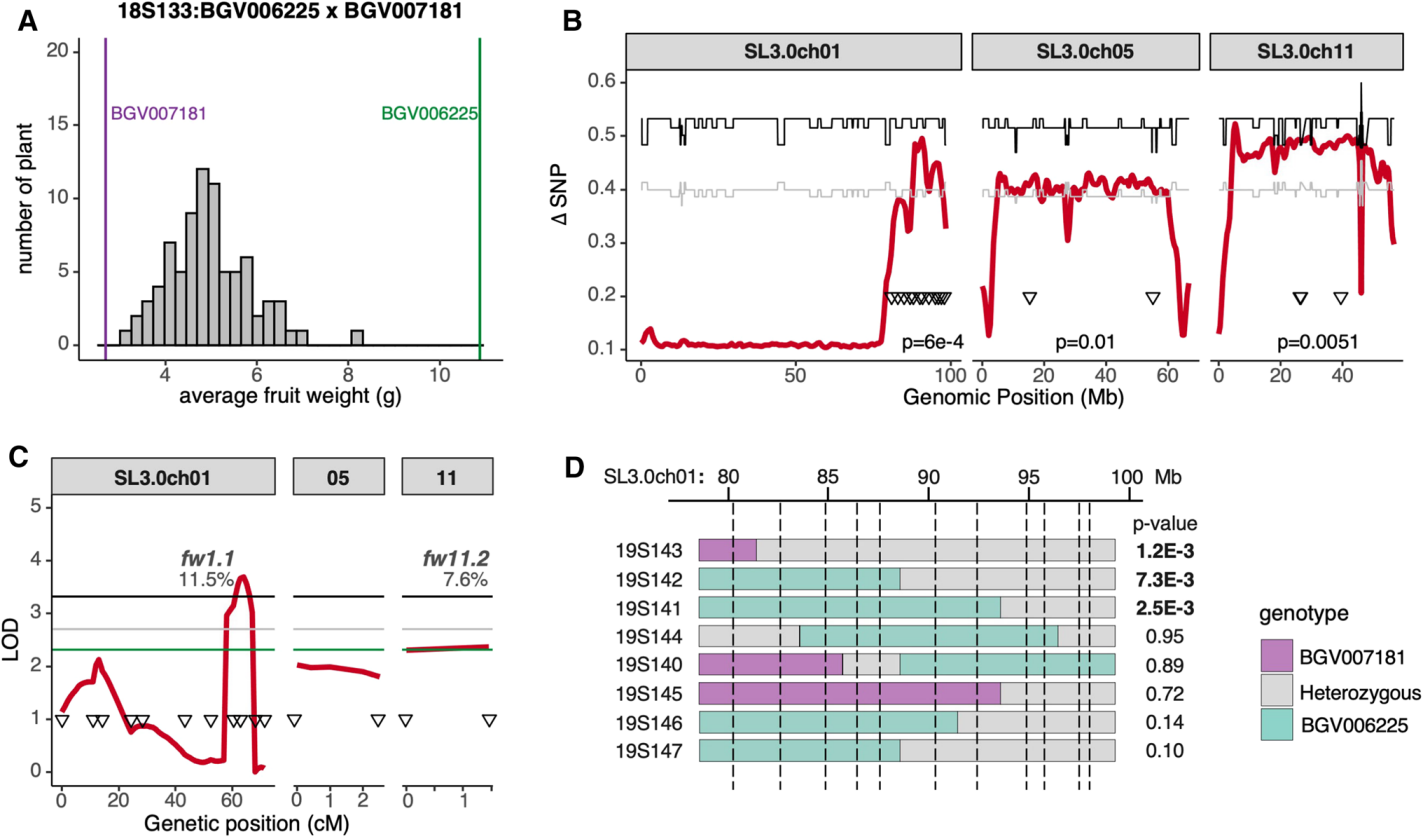
**A.** Fruit weight distribution of F_2_ plants in population 18S133. The vertical bars represent the fruit weight of parental accessions grown together with the F_2_ population. **B.** Fruit weight QTL-seq analysis of 18S133. Average ΔSNP values (red) in the 1 Mb sliding window are shown. Confidence interval at 95% (gray) or 99% (black) is represented in each plot. Genetic markers (triangles) were used to validate the QTL in the entire population. The p-values represent the correlation between the fruit weight and the most associated markers. **C.** Composite interval mapping of fruit weight in 18S133 using the indicated markers (triangles). LOD score (red) and confidence interval at 90% (green), 95% (gray) or 99% (black) are represented. 90% confidence interval was used due to the lower plant number in 18S133. The percentage of trait variation accounted by each QTL is shown. **D.** Fruit weight of F_3_ progenies segregating at the heterozygous regions was compared by Student’s *t* test. Dotted vertical lines mark the position of markers

The second population 18S133 (*n* = 81) was developed from SLC_ECU BGV006225 and SP_NECU BGV007181, weighing 10.9 g and 2.7 g, respectively (Fig. 6A and Table 1B). We observed higher pericarp area ratio in the large-fruited parent, which was caused by more cell layers and larger cell size in the pericarp tissue. Fruit weight QTLs in this population may have arisen during the first step of tomato domestication. The QTL-seq analysis of 18S133 revealed three new QTLs: a broad QTL on the bottom of chromosome 1 and two QTLs spanning the centromeric region of chromosome 5 and chromosome 11 (Fig. 6B, Supplementary Fig. 9B). Composite interval mapping using the entire F_2_ population confirmed the QTLs at chromosome 1 (*fw1.1*) and chromosome 11 (*fw11.2*) (Fig. 6C, Supplementary Table 4); they each accounted for 11.5% and 7.6% fruit weight variation in the population, respectively. The increase in fruit weight came from the SLC_ECU BGV006225 in both cases. We tested *fw1.1* in F_3_ families which were fixed at the centromeric *fw11.2* locus. The effect of *fw1.1* was validated in three families and narrowed down to an interval of 5.6 Mb, ranging from 92.9 Mb to the bottom of chromosome 1 (Fig. 6D, Supplementary Table 5). Therefore, the progeny testing of the locus supported the presence of a weight QTL albeit that the results were not conclusive as to the location of interval.

**Fig. 7:**
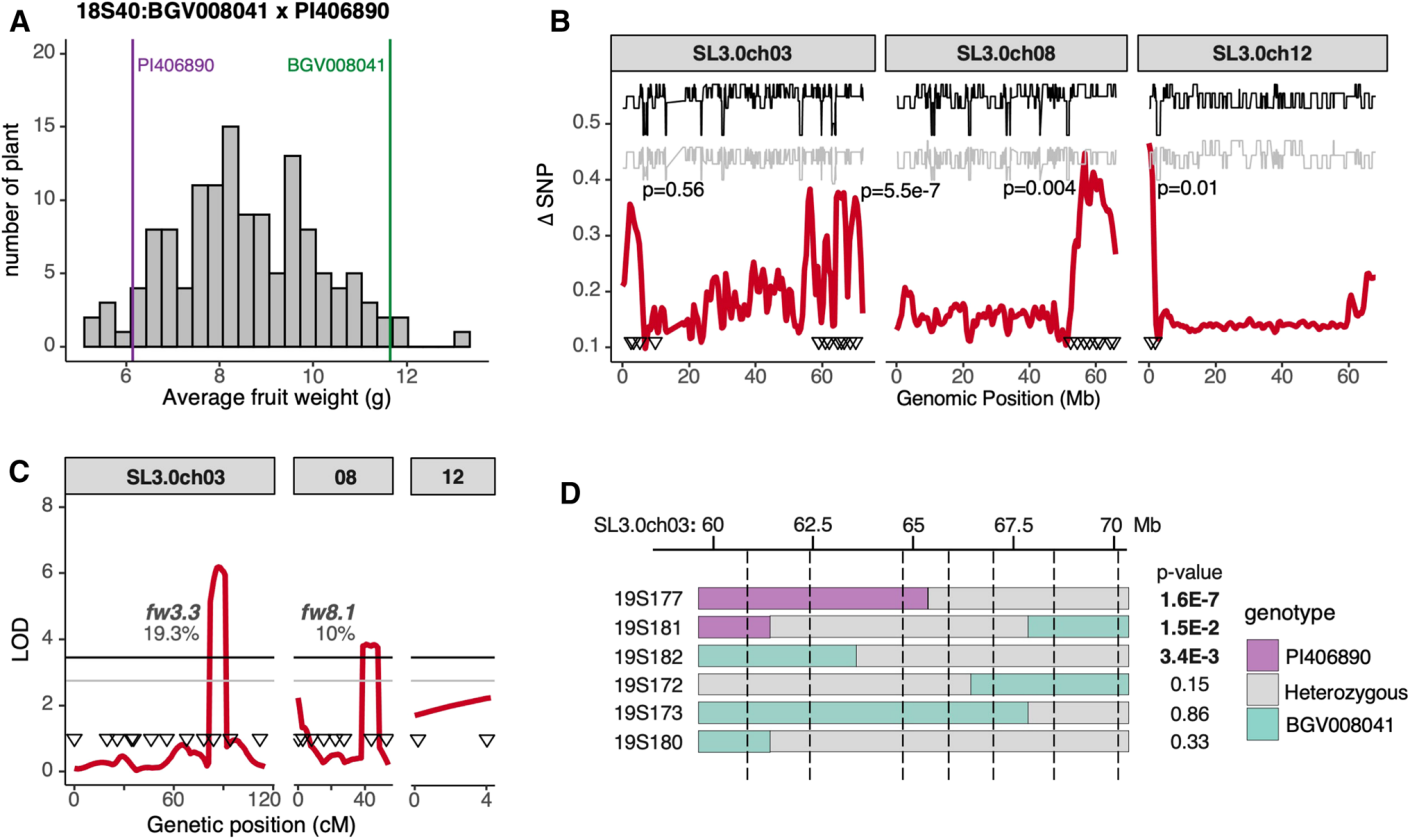
**A.** Fruit weight distribution of F_2_ plants in population 18S40. The vertical bars represent the fruit weight of parental accessions grown together with the F_2_ population. **B.** Results of fruit weight QTL-seq analysis in 18S40. Average ΔSNP values (red) in 1 Mb sliding window are shown. Confidence interval at 95% (gray) or 99% (black) is represented in each plot. Genetic markers (triangles) were used to validate the QTL in the entire population. The p-values represent the correlation between the fruit weight and the most associated markers. **C.** Composite interval mapping of fruit weight in 18S40 using the indicated markers (triangles). LOD score (red) and confidence interval at 95% (gray) or 99% (black) were represented. The percentage of trait variation accounted by each significant QTL is listed next to the QTL peak. **D.** Fruit weight of F_3_ progenies segregating at the heterozygous regions was compared by Student’s *t* test. Dotted vertical lines mark the position of markers

The last population 18S40 (*n* = 134) was developed from two closely related SLC_PER accessions, BGV008041 and PI406890, weighing 11.6 g and 6.1 g, respectively. The larger-fruited BGV008041 had pericarp twice as thick as PI406890, most likely due to larger cell size which was already established in the ovary during floral development (Table 1C). The fruit weight of the F_2_ population centered between the weight of parental accessions with a few individuals showing transgressive segregation (Fig. 7A). Much fewer SNPs differentiated these parents compared to the other populations due to the close relationship between BGV008041 and PI406890. Nevertheless, we identified three new QTLS: two QTLs on the bottom of chromosome 3 (*fw3.3*) and chromosome 8 (*fw8.1*) and a very narrow QTL on the top of chromosome 12 (*fw12.1*) (Fig. 7B, Supplementary Fig. 9C, Supplementary Table 4). Composite interval mapping confirmed *fw3.3* and *fw8.1* in the F_2_ population, and they accounted for 20% and 10% of fruit weight variation, respectively (Fig. 7C). The increase in fruit weight came from PI406890 and BGV008041, respectively. We tested the effect of *fw3.3* and *fw8.1* in progeny families segregating at one locus and fixed at the other (Fig. 7D, Supplementary Table 5). *fw3.3* was validated and delimited to the interval between 64.6 Mb and 68.4 Mb on chromosome 3 in three F_3_ families. *fw8.1* was not confirmed in the next-generation progeny testing. Interestingly, the PI406890 allele of *fw3.3* from the smaller-fruited parent increased fruit weight in this F_2_ population. Since *fw3.3* was the major QTL explaining 20% fruit weight variation, additional QTLs that may include *fw8.1* were likely to segregate in the population in an effort to counter the effect of *fw3.3* in the large-fruited parent.

In total, six fruit weight QTLs were identified of which three were validated in the next generation by progeny testing. We successfully avoided identifying the same QTLs in these three populations by selecting parents with distinctive cellular and developmental differences.

### Segregation of the novel fruit weight QTLs in other bi-parental populations

Next we sought to determine whether the novel fruit weight QTLs segregated in other F_2_ populations developed for this study. The genotype of the markers that were located in the QTL intervals showed that the six QTLs segregated in other populations (Table 2). *fw2.3* segregated in 11 populations, most of which were derived from crosses between SP and SLC_ECU. The other QTLs segregated in fewer populations with parents from more diverse background. At least one of the major QTLs (*fw1.1*, *fw2.3* or *fw3.3*) segregated in all populations except 18S36 and 18S37, supporting their importance during tomato domestication.

**Table 2:** Segregation of the newly identified fruit weight QTLs in twenty-one F_2_ populations

	Population	No. of F_2_ plants	Small-fruited	Large-fruited	Known genes*	*fw1.1*	*fw2.3*	*fw3.3*	*fw7.1*	*fw8.1*	*fw11.2*
SP_NECU x SLC_ECU	19S309	136	BGV007181	BGV006901	33,333	0.976	**0.024**	0.281	–	–	0.063
	18S92	134	BGV007181	BGV006768	33,333	0.258	**5.03E-05**	0.443	0.178	–	0.269
	18S129	126	BGV007181	BGV006779	33,333	0.070	**1.42E-05**	0.284	0.437	–	0.059
	18S90	128	BGV007181	BGV006792	33,333	0.821	**1.91E-05**	0.064	0.119	–	0.072
	18S133	81	BGV007181	BGV006225	33,333	**0.001**	0.318	0.669	0.301	**0.049**	**0.005**
SP_PER x SLC_ECU	18S141	141	BGV007198	BGV006768	33,333	0.149	**0.005**	0.833	0.523	–	0.281
	18S137	136	BGV007198	BGV006779	33,333	0.835	**6.50E-05**	**0.001**	0.156	–	0.971
	18S131	140	BGV007198	BGV006792	33,333	0.059	**5.82E-06**	0.491	0.048	–	0.105
	18S88	104	BGV007198	BGV006225	33,333	0.431	**0.009**	0.143	0.511	**0.049**	0.074
SLC_ECU x SLC CA/MEX	17S62	137	BGV007931	BGV006768	33,333	0.591	**3.46E-10**	**0.006**	**5.35E-05**	**0.010**	**3.63E-04**
	18S33	127	BGV007931	BGV006232	33,333	**0.019**	**0.026**	**0.004**	**0.002**	–	0.607
	18S36	129	BGV008108	BGV007023	33,333	0.059	0.082	0.494	–	**0.015**	**0.002**
	19S186	272	CATIE-11106	BGV006906	33,333	0.307	**6.27E-09**	0.298	–	–	0.508
	19S315	135	BGV007910	BGV006229	33,333	0.059	0.120	**0.036**	0.071	0.064	0.852
SLC x SLL	19S307	115	BGV008354	BGV007860	11,113	0.802	0.272	**0.001**	–	0.221	–
SLC x SLC	18S35	129	BGV006931	BGV014508	11,313	**8.37E-05**	0.107	**0.009**	–	0.483	**2.60E-06**
	19S185	131	BGV008225	BGV006232	33,333	**0.001**	0.307	0.812	–	–	–
	18S32	124	BGV007981	BGV013161	11,131	0.112	0.544	**1.57E-05**	–	–	0.485
	18S40	135	PI406890	BGV008041	11,333	0.584	0.770	**2.81E-07**	–	**0.004**	0.484
	18S37	142	BGV014516	BGV014515	13,313	0.659	0.407	0.381	–	–	–
	19S305	144	BGV008106	BGV014515	13,313	**0.038**	0.240	0.765	–	–	–

^*^Codification of the genotype at the known loci in the following order: *fw2.2, fw3.2, fw11.3, lc, fas*. The wild-type allele is coded as 3, and the derived allele as 1

## Discussion

### Fruit weight and related traits are highly quantitative and polygenic

Fruit weight is a quantitative trait with a complex genetic architecture. This complexity may be due to the multiple processes that accompany the increase of fruit weight during tomato growth. Weight can be partitioned into components related to morphology such as the pericarp, columella and septum areas, and components related to development such as cell number and cell size in the walls of the ovary and mature fruits. Each of these growth parameters may have undergone different selection pressures during the domestication and diversification of the crop. We showed that the septum and columella tissues contributed in higher proportions to fruit weight increases than pericarp tissue during tomato’s evolution. The data also showed that increased cell expansion impacted fruit size to a larger extent than increased cell number. The apparent importance of cell size increase in tomato domestication and diversification was also supported by genetic evidence: *CSR/fw11.3* (Mu et al. 2017) led to a larger increase in fruit weight than *CNR/fw2.2* (Frary et al. 2000) across the domestication gradient (Fig. 4B). Furthermore, the characterization of 20 tomato varieties showed a higher relative increase in cell size than in cell number with weight (Cheniclet et al. 2005). These findings suggest that the columella area and cell size increases were important drivers of fruit weight increases in tomato’s evolution.

The genetic architecture of fruit weight has been thoroughly studied in tomato resulting in the mapping of QTL and GWAS loci on every chromosome (Lin et al. 2014; Illa-Berenguer et al. 2015; Barrantes et al. 2016; Pascual et al. 2016). Yet most of these loci have not been further validated by progeny testing or finemapping. Here, we identified six fruit weight QTLs in three bi-parental populations and evaluated the presence of these QTL in additional F_2_ populations. The percentage of variation explained by these QTLs ranged from 10 to 25%. In each population, the progeny testing using F_3_ families worked best for validating a major QTL, such as *fw2.3* that accounted for 25% fruit weight variation. This locus was also confirmed in many other segregating families. By contrast, *fw1.1*, explaining only 11.5% variation, was not consistently validated by progeny testing in the next generation. *fw1.1* was also found in just three additional segregating F_2_ families. Most F_2_ populations segregated for one or two QTLs, leaving still a high proportion of the phenotypic variation unexplained. Environmental conditions affect fruit weight to some extent, yet additional QTLs from these populations likely remain. Importantly, according to the omnigenic theory proposed to explain the molecular genetic bases of complex traits (Boyle et al. 2017; Liu et al. 2019), certain major QTLs can be considered core genes. Core genes are thought to have a direct effect on the phenotype. But many other QTLs underlying genes that are expressed only in certain cell types can be considered peripheral genes. Peripheral genes make small contributions to the phenotype possibly through regulatory networks. In addition to the known tomato fruit weight genes, some of the newly identified QTLs might be core genes, especially if they segregate in multiple F_2_ populations (Table 1). Other QTLs may have minor roles in fruit weight regulation and can be considered peripheral genes. In addition, many other peripheral genes remain unidentified possibly due to their small effect on the phenotype. Since the phenotypic variance that is not explained is rather large in these populations, the likelihood of many small effect QTL is high. The discovery of minor genes would require advanced experimental design (complex multi-parent populations or increased number of sample size), as well as selectively fixing all core genes to avoid their interference.

### Evolution of the fruit weight genes and QTLs in tomato populations

**Fig. 8:**
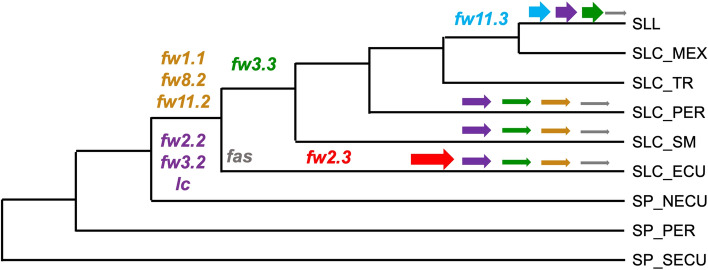
Schematic representation of the origin of the derived alleles of the QTLs (symbols) and how these alleles were selected throughout tomato’s domestication (arrows). The width of the arrow suggests the relevance/allele frequency within a subpopulation. The origin of the subpopulations was inferred from Razifard et al. (2020)

The Varitome collection and many F_2_ populations spanned four key steps in tomato domestication and diversification history: (1) the transition from fully wild SP to the semi-domesticated SLC_ECU, (2) the diversification of SLC in South America, (3) the migration northward and the potential de-domestication to wild-like SLC in transitional SLC and (4) the transition from semi-domesticated to early domesticated SLL in Mexico (Razifard et al. 2020). We estimated where and when fruit weight improvement alleles may have arisen and whether they were selected (Fig. 8).

1. The rise of derived alleles of *fw2.2*, *fw3.2*, *lc* and newly identified *fw2.3* was most relevant in the fruit weight increase during the first step of tomato’s evolution from SP to SLC. The derived alleles of the known genes were found at intermediate frequencies in SLC_ECU. The combination of these four alleles in the South American SLC caused a significant increase in fruit weight (Fig. 4B). *fw2.3* segregated in eight out of nine SP x SLC_ECU populations and the SLC_ECU accessions carried the derived allele contributing to larger fruits. In addition, *fw1.1*, *fw8.1* and *fw11.2* segregated in one SP_NECU x SLC_ECU population, and *fw3.3* and *fw8.1* segregated each in one SP_PER x SLC_ECU population, suggesting that the derived alleles of these QTLs also appeared in SLC_ECU but were present at lower allele frequencies.
2. The diversification of SLC in South America was shaped by *fw1.1*, *fw3.3, fw8.1* and *fw11.2*, in addition to the known genes already found in SLC_ECU. The allele frequencies of the derived alleles for these genes increased in SLC_PER and SLC_SM. Furthermore, *fw1.1* and *fw3.3* segregated in two and three F_2_ populations between SLC_ECU, SLC_PER and SLC_SM, whereas *fw8.1* and *fw11.2* segregated only in one population. In most cases, SLC_ECU carried the derived allele contributing to larger fruits, except in SLC_SM x SLC_ECU populations. This suggests that SLC_SM may carry a second, more favorable derived allele. *fw2.3* did not segregate in populations developed from South American SLC, which may indicate that the derived allele arose in SLC_ECU and was fixed in most SLC from South America.
3. Derived alleles in South American SLC contributing to larger fruits may not be present in SLL. The SLC_MEX is genetically most related to SLL, yet the transitional SLC bore smaller fruits than South American SLC (Fig. 2). The frequency of derived alleles for known fruit weight genes was also reduced, consistent with a reduction in fruit weight (Fig. 4). All QTLs segregated in at least one SLC_ECU x SLC_TR/MEX, suggesting that they all played a role in the fruit weight decrease during the northward migration of SLC. Interestingly, certain transitional SLC carried the WT allele for all six fruit weight QTLs in the analyzed populations, which supported the hypothesis that favorable derived alleles contributing to larger fruits were left behind during domestication.
4. The last transition from SLC to SLL in Mexico was primarily explained by *fw11.3* and *fw3.3*. The derived allele of *fw11.3* appeared at low frequencies in SLC and was nearly fixed in SLL, correlating with a two-fold increase on average in fruit weight. In addition, *fw3.3* segregated in the SLC x SLL population contributing to an increase of 18 g in fruit weight.

In summary, most of the derived alleles for both known fruit weight genes and newly identified QTLs seemed to have arisen within the South American SLC subpopulation, mainly in Ecuador. Therefore, the South American SLC may carry several derived alleles for fruit weight QTLs absent from Central American SLC and SLL and indeed available to be introgressed into modern SLL.

## Supplementary Information

Below is the link to the electronic supplementary material.

Supplementary file1 (XLSX 68 KB)

Supplementary file2 (JPEG 235 KB)

Supplementary file3 (JPEG 314 KB)

Supplementary file4 (JPEG 115 KB)

Supplementary file5 (JPEG 725 KB)

Supplementary file6 (JPEG 229 KB)

Supplementary file7 (JPEG 294 KB)

Supplementary file8 (JPEG 221 KB)

Supplementary file9 (JPEG 188 KB)

Supplementary file10 (JPEG 498 KB)

## Data Availability

All relevant raw data are available as supplementary material and/or upon request.
