# Supplementary figures and images for: Unraveling the genetics of tomato fruit weight during crop domestication and diversification
Source: Theor Appl Genet. 2021 Jul 12;134(10):3363–78. doi: 10.1007/s00122-021-03902-2 (PMC8440300; doi:10.1007/s00122-021-03902-2)

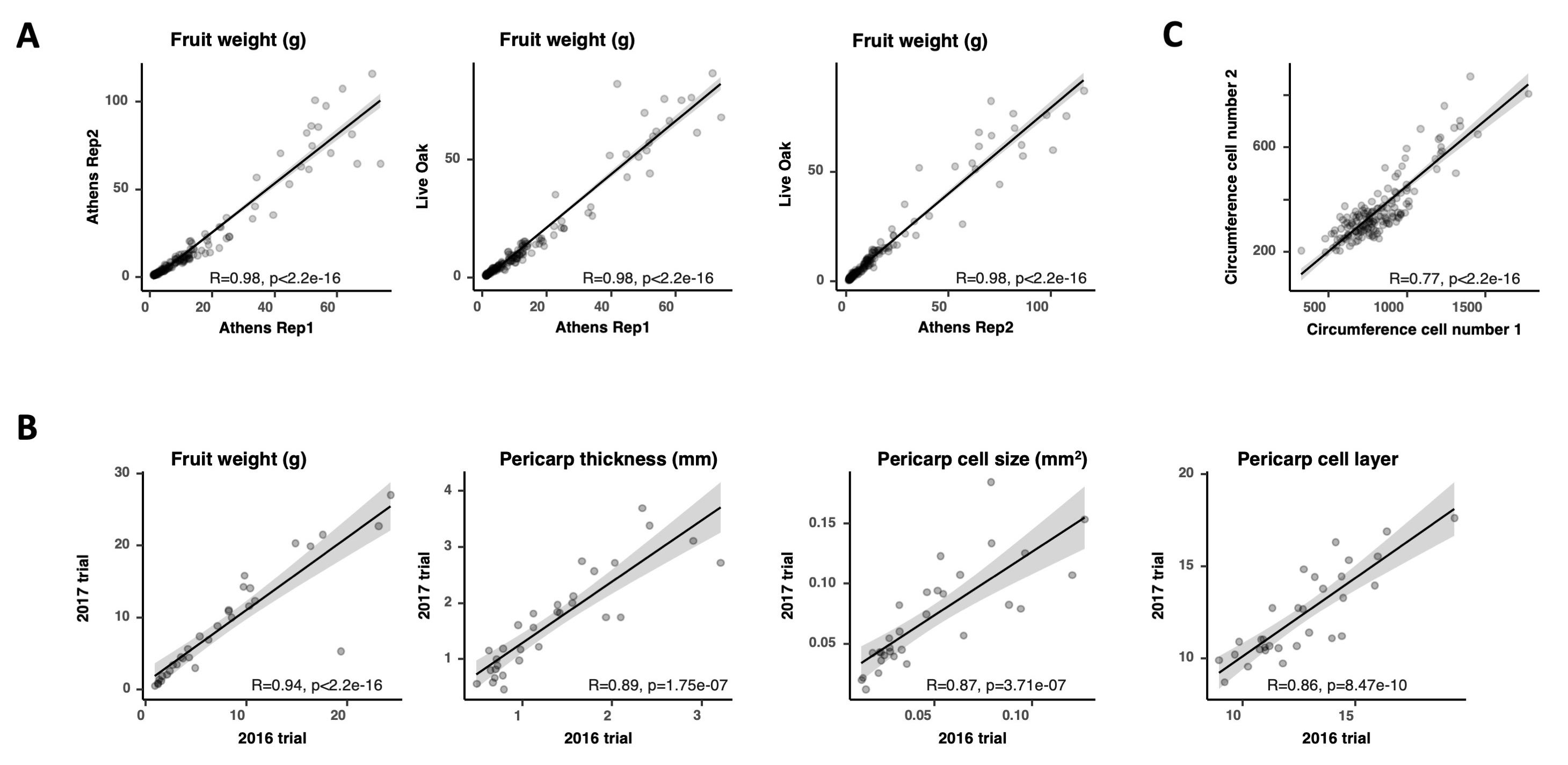

Supplement: Supplementary file 2 — Supplementary file2 (JPEG 235 KB) [file 122_2021_3902_MOESM2_ESM.jpeg]

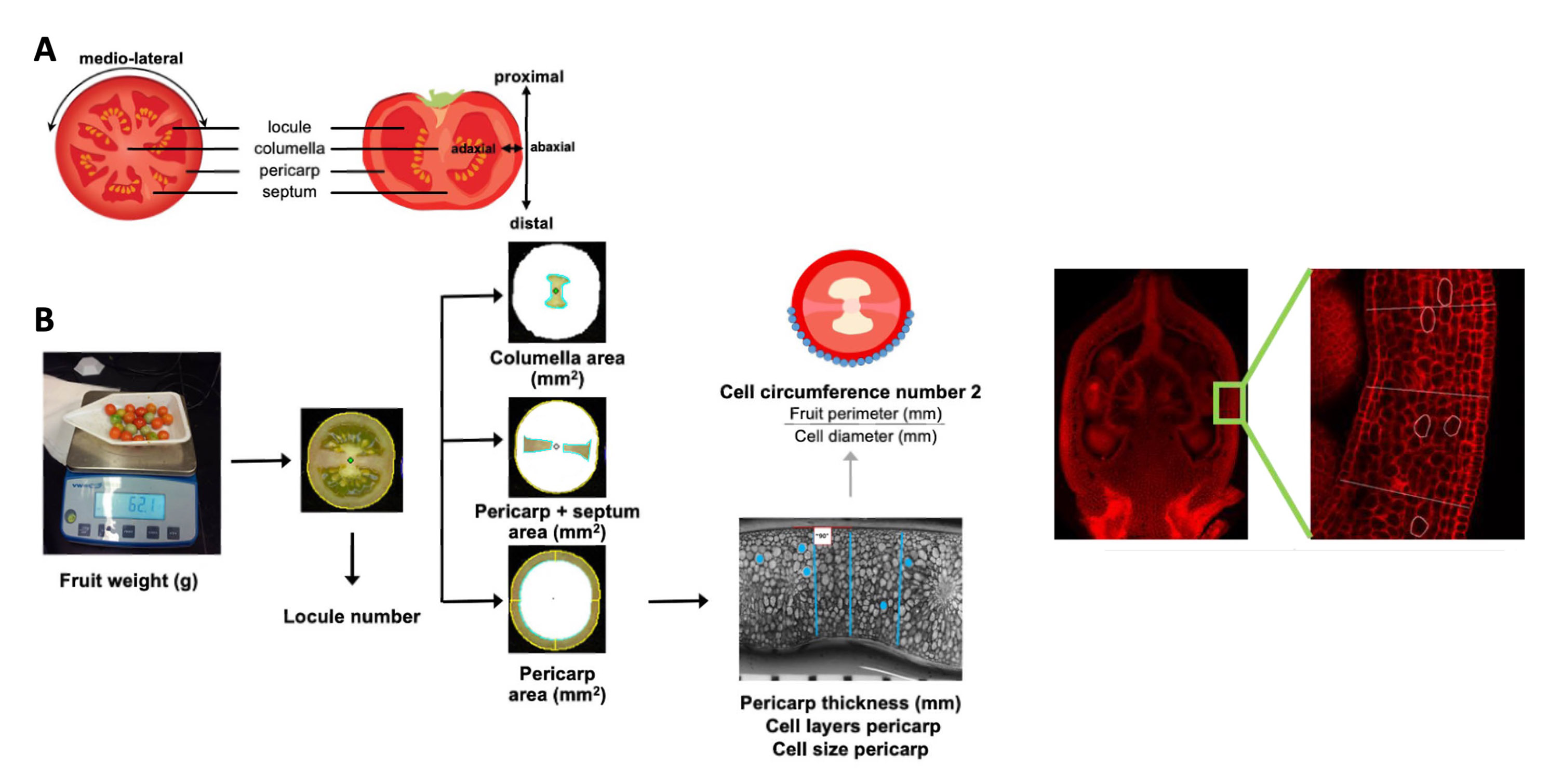

Supplement: Supplementary file 3 — Supplementary file3 (JPEG 314 KB) [file 122_2021_3902_MOESM3_ESM.jpeg]

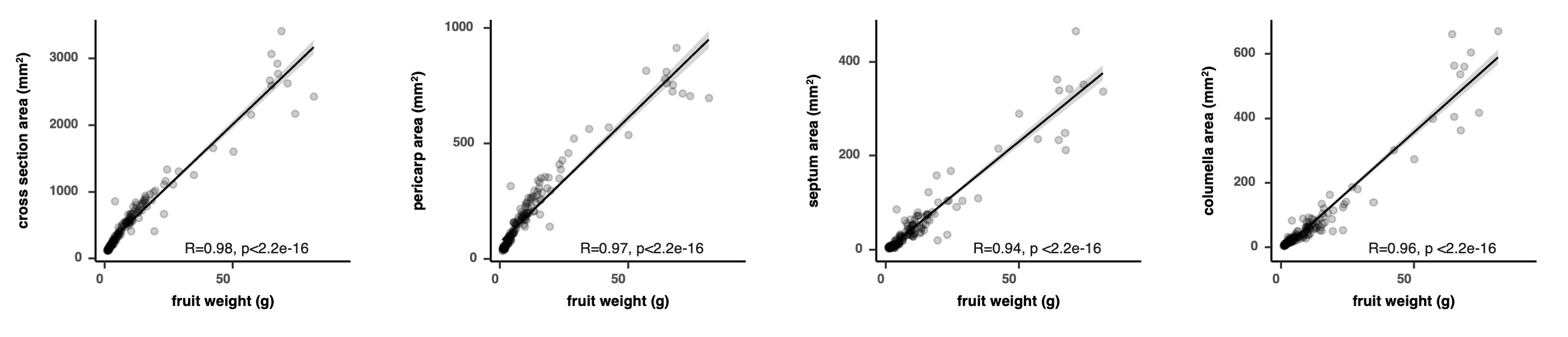

Supplement: Supplementary file 4 — Supplementary file4 (JPEG 115 KB) [file 122_2021_3902_MOESM4_ESM.jpeg]

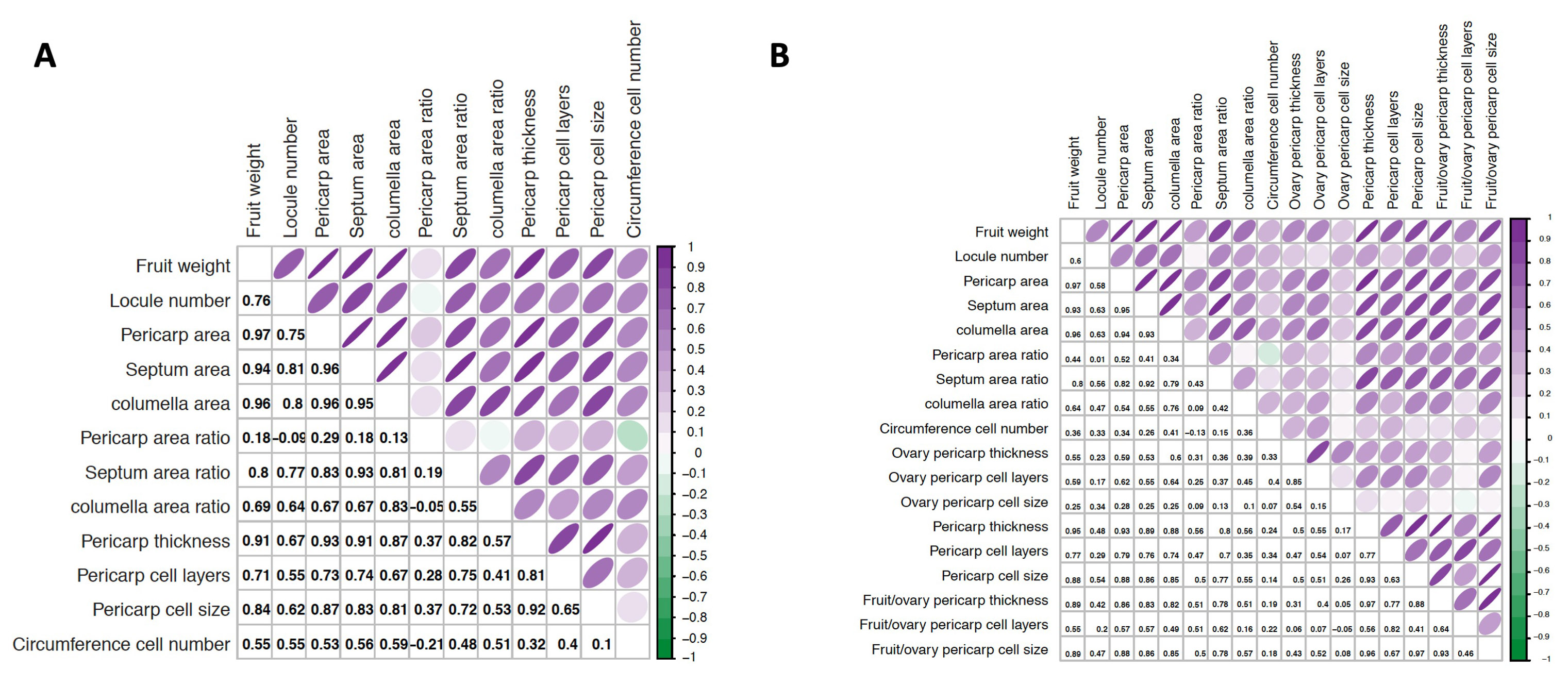

Supplement: Supplementary file 5 — Supplementary file5 (JPEG 725 KB) [file 122_2021_3902_MOESM5_ESM.jpeg]

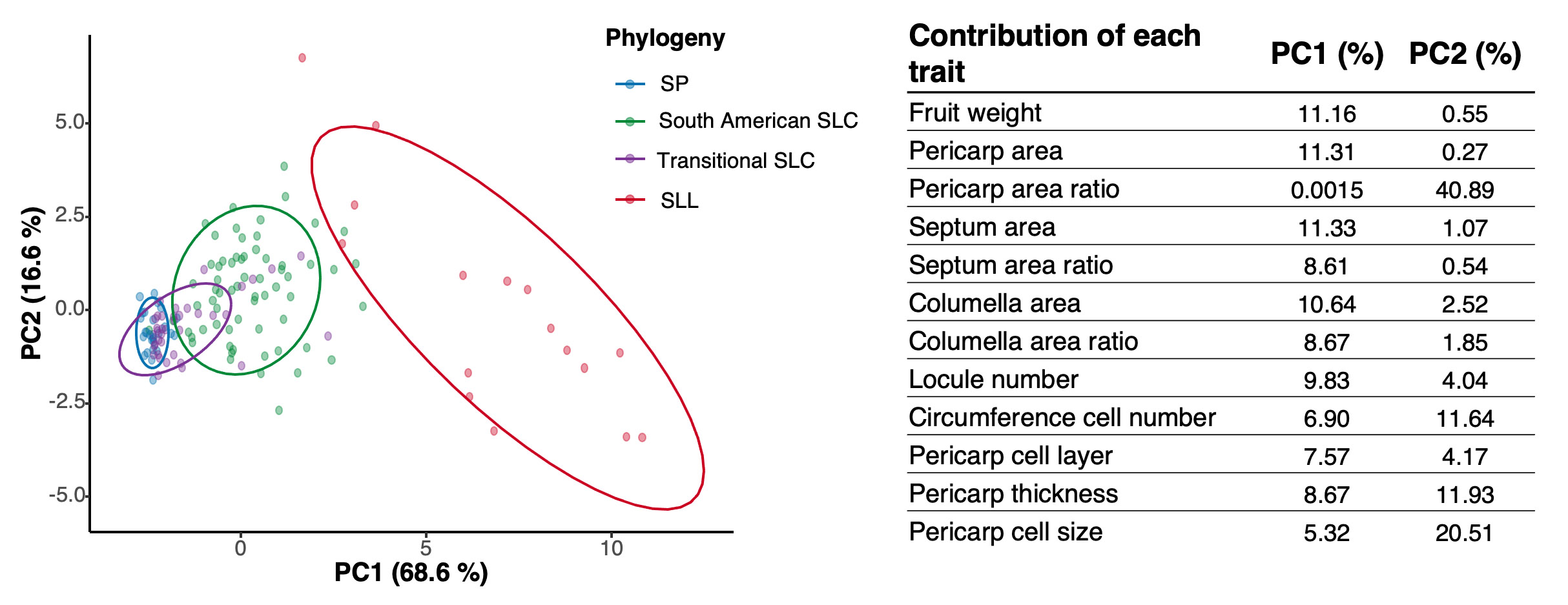

Supplement: Supplementary file 6 — Supplementary file6 (JPEG 229 KB) [file 122_2021_3902_MOESM6_ESM.jpg]

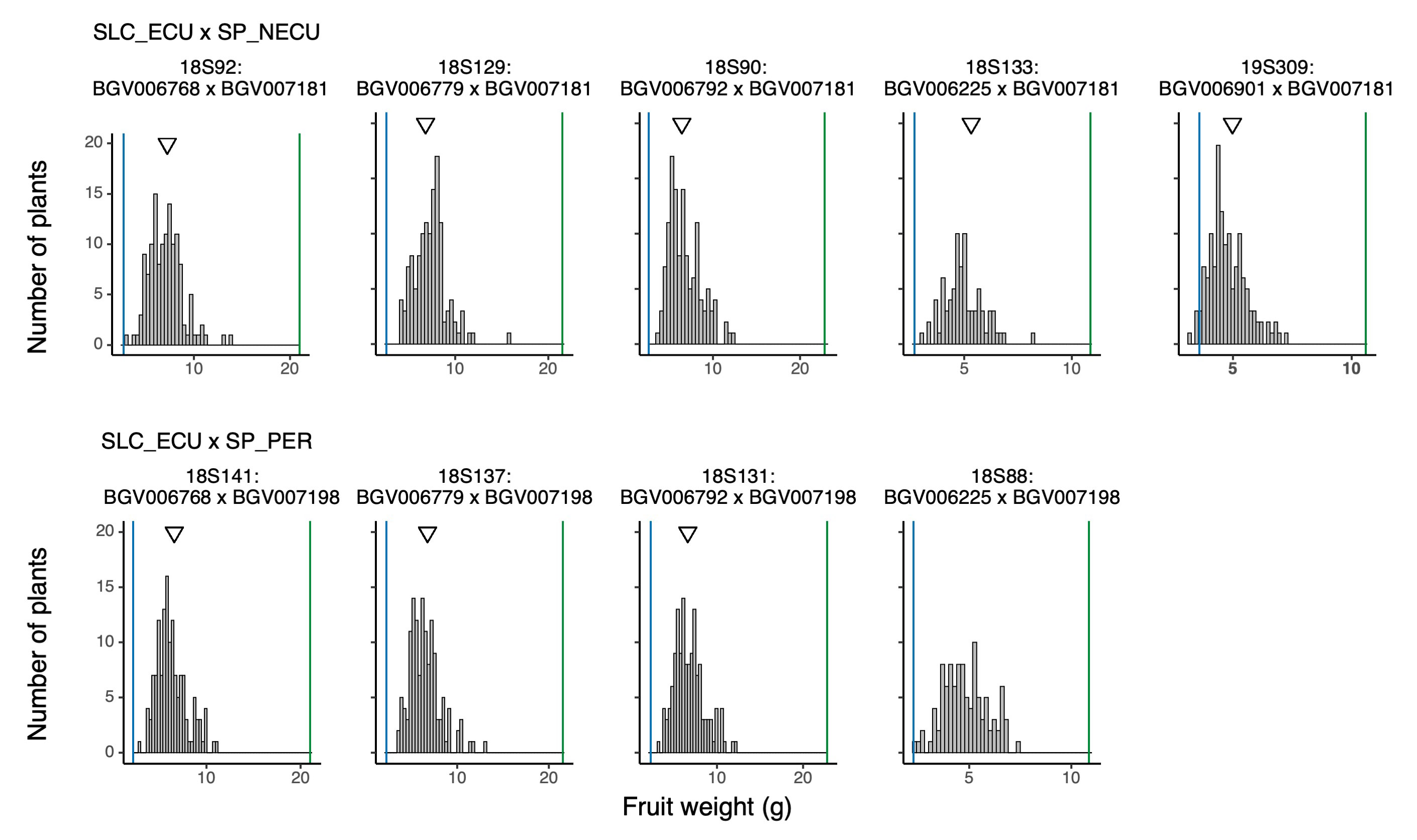

Supplement: Supplementary file 7 — Supplementary file7 (JPEG 294 KB) [file 122_2021_3902_MOESM7_ESM.jpeg]

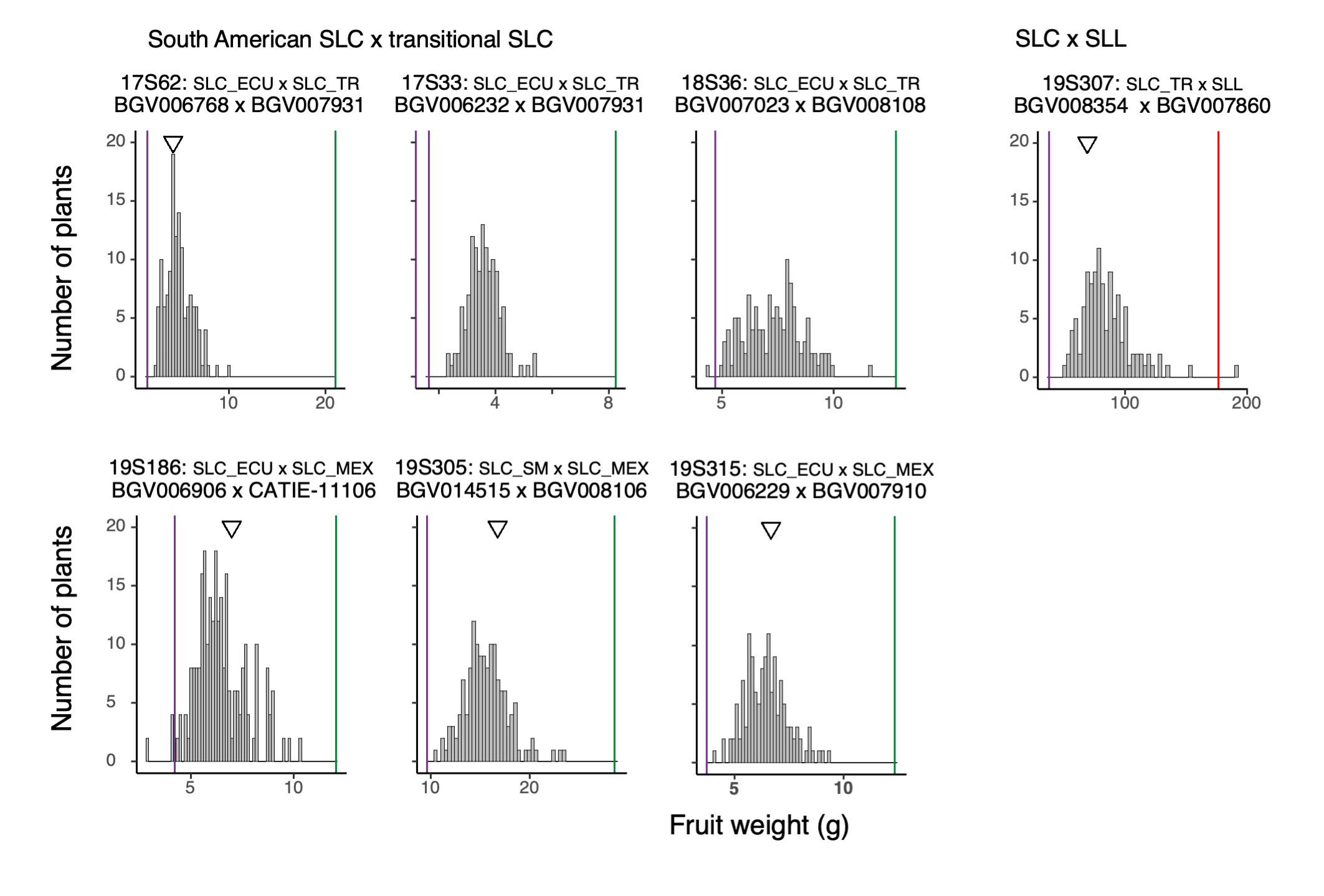

Supplement: Supplementary file 8 — Supplementary file8 (JPEG 221 KB) [file 122_2021_3902_MOESM8_ESM.jpg]

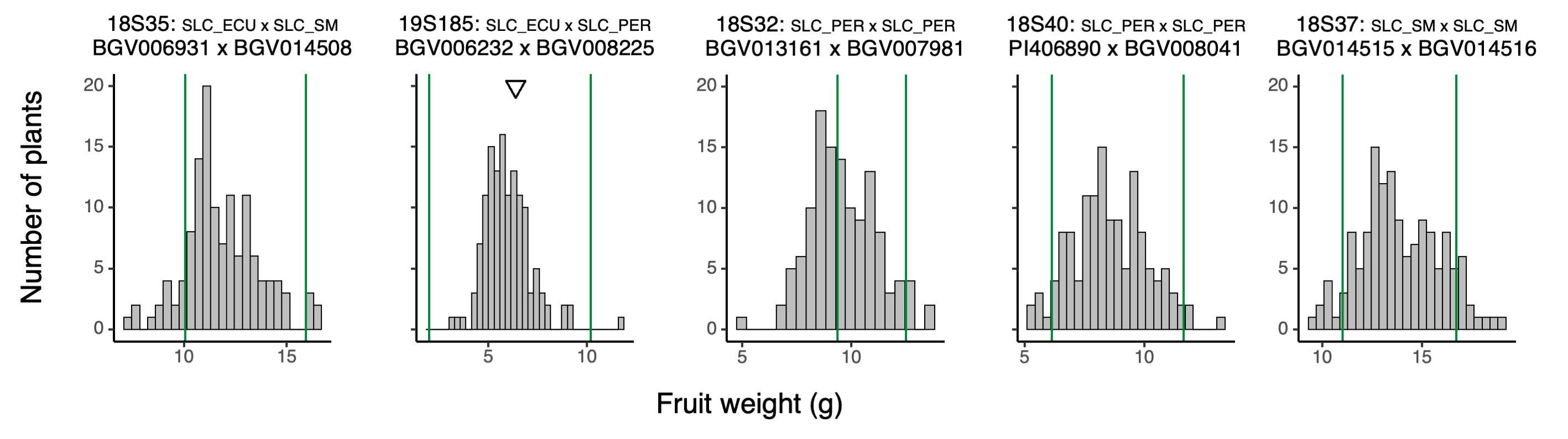

Supplement: Supplementary file 9 — Supplementary file9 (JPEG 188 KB) [file 122_2021_3902_MOESM9_ESM.jpeg]

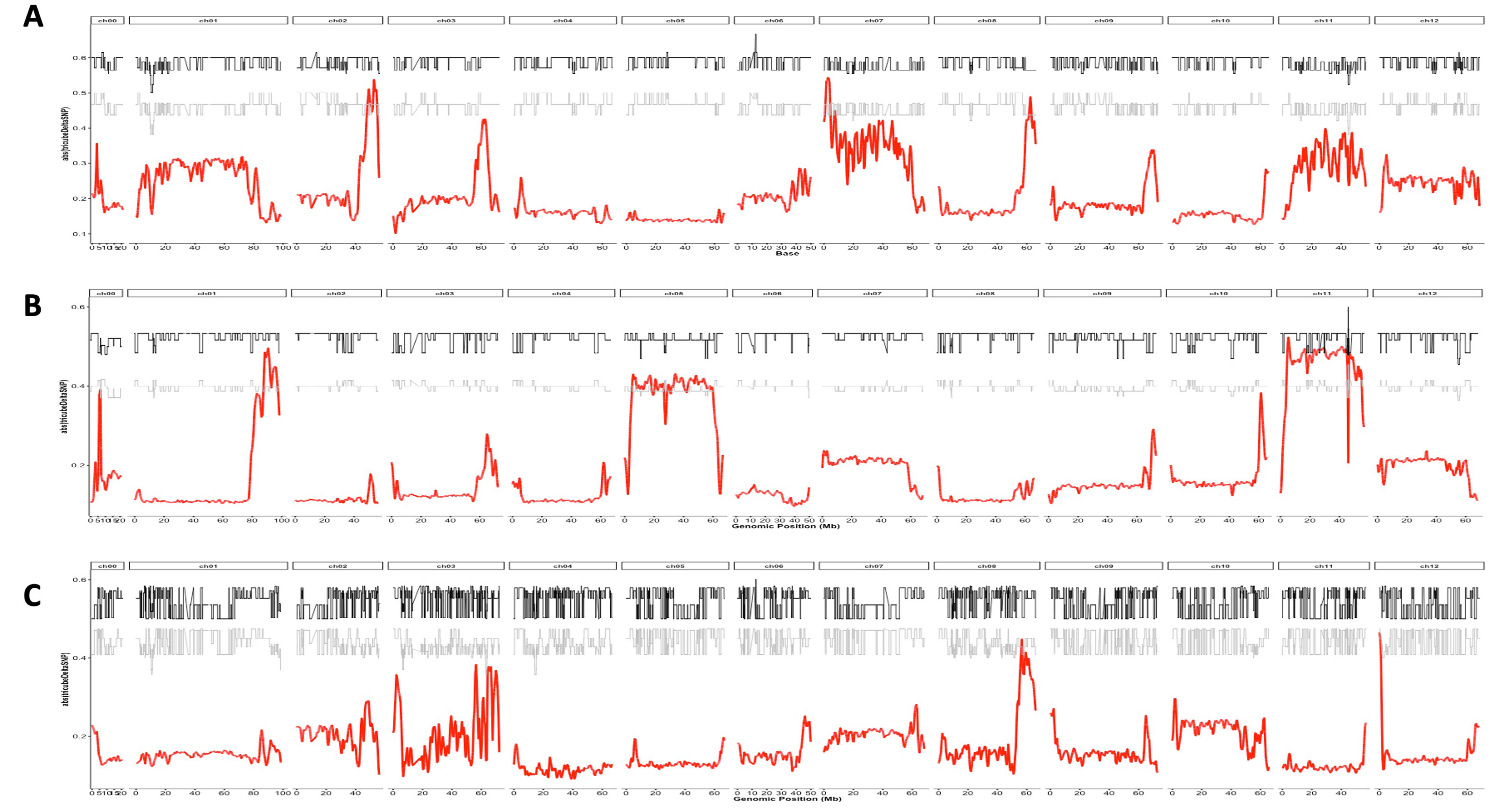

Supplement: Supplementary file 10 — Supplementary file10 (JPEG 498 KB) [file 122_2021_3902_MOESM10_ESM.jpeg]
